# Ensemble machine learning prediction model for clinical refraction using partial interferometry measurements in childhood

**DOI:** 10.1371/journal.pone.0328213

**Published:** 2025-07-10

**Authors:** Sa Ra Kim, Dong Hyun Kang, Gon Soo Choe, Dae Hee Kim

**Affiliations:** 1 Department of Ophthalmology, Kim’s Eye Hospital, Seoul, Korea; 2 Data Center, Kim’s Eye Hospital, Seoul, Korea; Hangil Eye Hospital / Catholic Kwandong University College of Medicine, KOREA, REPUBLIC OF

## Abstract

**Purpose:**

To develop an ensemble machine learning prediction model for clinical refraction in childhood using partial interferometry measurements.

**Methods:**

Age, sex, cycloplegic refraction, and partial interferometry data collected within one month were obtained from patients aged 5–16 years, retrospectively. Four ensemble regression models were used to develop prediction models of spherical equivalents (SE) from the collected data. Root mean squared error (RMSE) was used to compare the accuracy among the models. The accuracy of the ensemble models was compared with that of a previously developed multiple linear regression model.

**Results:**

4156 eyes from 1965 patients (50.3% female) were included. Mean age was 8.4 ± 2.3 years and mean SE was −1.01 ± 2.94 diopters. Mean axial length was 23.63 ± 1.41 mm and mean keratometry reading of flat and steep axis was 43.58 ± 1.40 diopters. Developed ensemble models had accuracy of RMSE 0.800 to 0.829 diopters, which was superior to that of the conventional regression model (1.213 diopters). Simulations with the same biometric parameters showed that female sex was associated more with myopia than that of male sex. Long eyes showed dampened increase in the myopic refraction per unit axial length.

**Conclusions:**

Refractive errors can be calculated in the childhood using these ensemble models with ocular biometric parameters. Moreover, the models were able to simulate hypothetical relationships between ocular parameters and SE to understand the nature of clinical refraction.

## Introduction

The clinical refraction test is an essential and basic test for ocular examination. Since manual retinoscopy was first invented for measuring clinical refraction in the late 19^th^ century, the technique has undergone much improvement. Thus far, cycloplegic refraction is the standard for measuring the refractive error [1]. However, manual retinoscopy has many limitations. Manual retinoscopy largely depends on the examiner’s skill; i.e., the measurements may vary depending on the examiner (intra- and interexaminer variability). One study reported the variability in the spherical and cylindrical error to be −0.85–0.85 diopters (D) and −0.68–0.62 D for intraexaminer evaluation, and −0.92–0.76 D and −0.75–0.59 D for interexaminer evaluation (95% limit of agreement), respectively, for manual retinoscopy [2]. Moreover, the examinee’s cooperation is essential for accurate retinoscopy results, as the examinee should fixate on a distant target to relax accommodation. Measurement errors in retinoscopy tests are therefore inevitable among children, even with the use of cycloplegia. Furthermore, pharmacologic cycloplegia requires a minimum of 30 minutes to elicit cycloplegia on examinees; however, a manual cycloplegic retinoscopy is a time-consuming test with relatively low precision.

The ideal clinical refraction test is an optical model based on ocular structural metrics and refractive index. Ocular refractive structural data include data regarding the cornea (topography, thickness, and curvature), anterior chamber, aqueous humor, crystalline lens, vitreous body, and retinal surface irregularity. With technological advancements in ocular biometric devices, metric data of ocular structures — such as corneal curvature, axial length, anterior chamber depth, crystalline lens curvature/thickness, and vitreous cavity depth — can be acquired. Partial interferometry has been used to measure reliable and reproducible ocular biometrics, even in young patients [3]. However, the ocular refractive index is an individual characteristic, and a method to measure the *in-vivo* refractive index is lacking. Accordingly, the calculation of clinical refraction from ocular structural data includes many assumptions for refractive indices of the ocular media.

Machine learning technology is widely used to make prediction models from previously observed data [4]. Nonlinear and self-adaptive modeling using machine learning technology, which compensates for unmeasured data by providing automatic and mathematical classifications of given data, makes it easier to develop an accurate prediction model for complicated, real-world problems. As refractive errors continuously change with ocular structural change throughout childhood [5], clinical refraction calculations from structural data involve complicated mathematical problems that can be improved using machine learning technology. Previous studies have investigated developing models to predict subjective refraction using wave-front aberrometry results [6,7].

This study was conducted to develop an ensemble machine learning prediction model for clinical refraction in childhood using partial interferometry measurements to acquire reliable and precise refractive errors from children without cycloplegia.

## Methods

The medical records from March 2017 to January 2020 were retrospectively reviewed. We subsequently included patients aged 5–16 years who underwent both a cycloplegic refraction and partial interferometry test with a 1-month interval between the two tests. We excluded patients who had undergone previous myopia suppression treatments, such as atropine or orthokeratology; and with congenital ocular malformations, such as anterior segment dysgenesis; ocular colobomatous diseases, congenital cataracts affecting visual acuity or aniridia; and corneal diseases, like corneal opacity or keratoconus. The study protocol conformed to the tenets of the Declaration of Helsinki. This study involved human participants, and was approved by the institutional review board (KEH 2022-01-005). The requirement for informed consent was waived owing to the use of retrospectively collected clinical data. The data were accessed from January 24, 2022 and analyzed anonymously.

The administration of cycloplegic eyedrops included one drop of 1% cyclopentolate hydrochloride (Cyclogyl^®^; Alcon Inc., Fort Worth, TX, USA), followed by three drops of 1% tropicamide (Mydriacyl^®^; Alcon Inc.) at 10-minute intervals. Cycloplegic retinoscopy was performed 45 minutes after the first instillation of cycloplegic eyedrop by an independent examiner, and repeated and confirmed by a single examiner (DHK) to minimize interexaminer variability. Partial interferometry was performed using the IOL master 500^®^ (Carl Zeiss Meditec, Jena, Germany). All partial interferometry measurements were reliably obtained considering the patient’s fixation status, signal-to-noise ratio (>100), and previous biometry data, if available. Unreliable measurements were reconfirmed by repeated measurements. We also collected demographic features, such as age and sex. Spherical and cylindrical errors were collected from cycloplegic refraction data. Axial length and keratometry readings (in D) at the flat and steep axis were collected from partial interferometric data.

Machine learning modeling included four supervised ensemble regression models, including Random Forest (RF) [8], Gradient Boosting Machine (GBM) [9], eXtreme Gradient Boost (XGB) [10], and Light Gradient Boosting Machine (LGBM) [11] regressions. The ensemble regression model included two sequential processes; first, computerized algorithms classified input data into several categories (a classified node), minimizing the variance of the same categorized data. This process can be performed by several branching decision trees (bagging; bootstrap aggregating, as in RFs) or other stepwise weighted classifiers (boosting, as in GBM, XGB and LGBM). Next, regression coefficients between independent variables and a dependent variable were calculated for each classified node.

We assumed total refractive errors as a function of ocular axial length and corneal refractive errors. We used spherical equivalents (SEs) (spherical error + 0.5 × cylindrical error) as dependent variables. For independent variables, axial length, flat and steep keratometry readings, and mean and difference of the keratometry readings were used for multivariable modeling. As the ocular refractive index may be affected by age and sex and cannot be directly acquired, age and sex were appended as independent variables [12].

Data were split into 80% for training, 10% for validation, and 10% for testing. Hyperparameters of the four ensemble models were tuned using grid search with ten-fold cross-validation conducted. The predefined parameter grid included the number of estimators (ranging from 100 to 1000 in increments of 100) and maximum depth (3, 5, 7, and from 9 up to 40). The best hyperparameter combination was selected based on the highest average F1-score across the cross-validation folds. After tuning, the final models were retrained on the entire training set using the optimal parameters and then evaluated on an independent test set.

To compare performances between the conventional linear regression and machine learning models, we adopted a multiple linear regression model from our previous study. The study used axial length and mean keratometry readings from the IOL Master 500^®^ for the prediction of the corresponding SE [13]. In this study, the SE was calculated as follows:


SE = 84.48 – 2.116 × (axial length [mm]) – 0.828 × (mean\ keratometry\ reading [D]) (1)

Statistical analyses and model construction were performed using Python version 3.7.9. Scikit-learn library (version 0.23.2) was used for RF and GBM regressions. XGB regression was performed using XGBoost library version 1.5.0. LGBM regression was performed using LightGBM library version 3.2.1. The total dataset was divided into training (90%) and test (10%) sets for the validation of the generated model. For accuracy assessments, the root mean squared error (RMSE) was used and expressed as follows:


$$\sqrt{\frac{\sum_{i=1}^{n}{\left(x_{1i}-x_{2i}\right)}^{2}}{n}}$$ (2)

where was $x_{1}\;$is designated as a predicted estimate of the SE, $x_{2}$ as the real SE value, and n as the total number of data. The importance of the independent variables was calculated using the built-in feature importance function.

## Results

**Fig 1:**
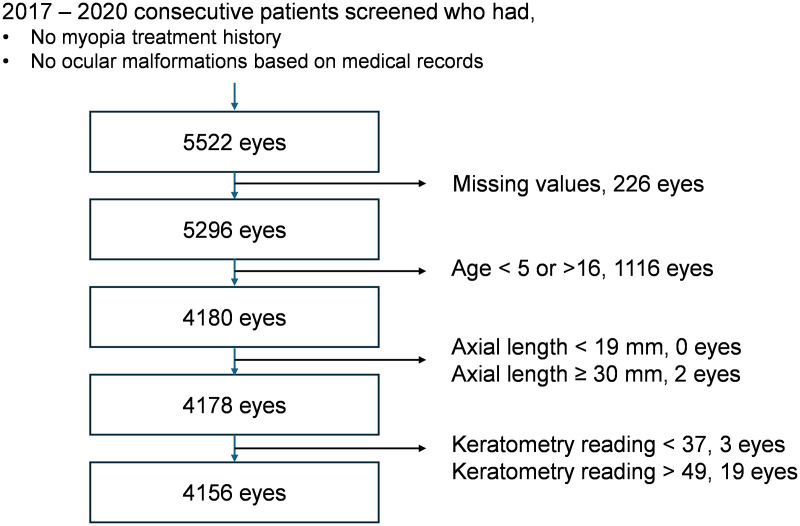
Flowchart of included patients. Among the 5522 eyes screened, 4156 were included, excluding those with missing values and outliers.

**Fig 2:**
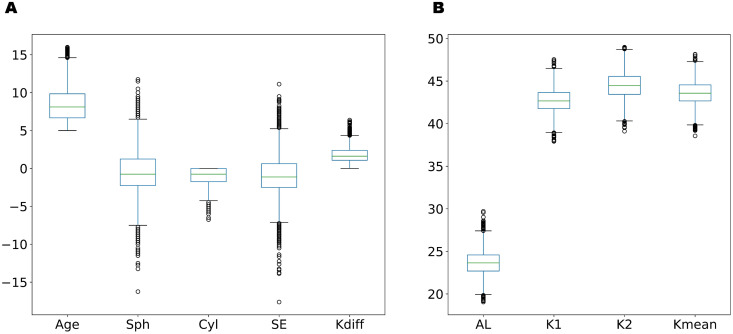
Boxplots of features included for modeling. Sph, spherical error; Cyl, cylindrical error; SE, spherical equivalent; Kdiff, the difference of the keratometry reading; AL, axial length; K1, flat K; K2, steep K; Kmean, mean of keratometry reading.

A total of 4156 eyes from 1965 patients were selected, including 988 females (50.3%; Fig 1). The mean age of the patients was 8.4 ± 2.3 (range: 5.0–16.0) years. The mean SE was −1.01 ± 2.94 (range: −17.625–11.125) D, and the mean axial length was 23.63 ± 1.41 (range: 19.05–29.07) mm. The mean keratometry reading of the flat and steep axis was 43.58 ± 1.40 (range: 38.56–48.16) D (Table 1, Fig 2).

**Table 1:** Baseline characteristics of participants.

		Mean	SD	Min	Max	IQR
**Age (years)**		8.4	2.3	5	16	3.2
**Cycloplegic refraction data (diopters)**	Spherical errors	−0.46	2.97	−16.25	+11.75	3.50
	Cylindrical errors	−1.11	1.13	−6.5	0.00	1.75
	Spherical equivalents	−1.01	2.94	−17.625	+11.125	3.125
**Biometry data**	Axial length (mm)	23.63	1.41	19.05	29.70	1.89
	Flat K (diopters)	42.67	1.38	37.92	47.54	1.89
	Steep K (diopters)	44.48	1.57	39.11	48.98	2.11
	Mean K (diopters)	43.58	1.40	38.56	48.16	1.88
	K difference (diopters)	1.81	0.99	0	6.38	1.31

Abbreviations: SD, standard deviation; K, keratometry reading; Min, minimum; Max, maximum; IQR, interquartile range

**Fig 3:**
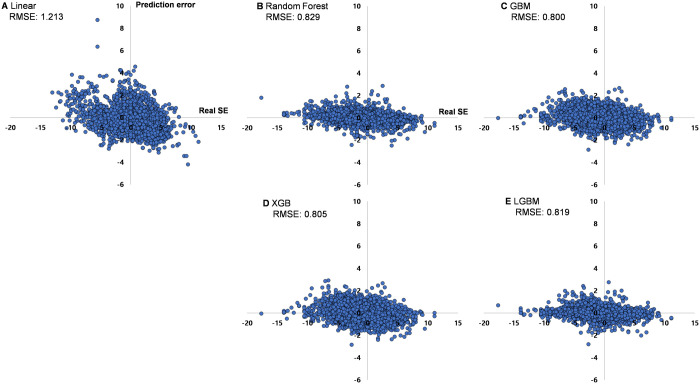
Prediction error (predicted spherical equivalents (SE) – real SE) according to real SE. (a) conventional multiple linear regression model, (b) random forest model, (c) GBM model, (d) XGB model, (e) LGBM model. Root mean squared error (RMSE) is indicated. Ensemble models were superior to conventional multiple linear regression model.

The accuracies of each model were similar. The RMSEs for each machine learning model were 0.829, 0.800, 0.805, and 0.819 for RF, GBM, XGB, and LGBM regressions, respectively; this meant that the predicted SE demonstrated an approximately 0.8 D difference from the real SE. Means (and 95% confidence intervals) of deviations between the predicted and real SEs for the test set were 0.059 (−0.026–0.145) D, 0.040 (−0.039–0.118) D, 0.032 (−0.049–0.112) D, and 0.052 (−0.028–0.133) D for RF, GBM, XGB, and LGBM regressions, respectively. Conversely, the RMSE for the previous multiple linear regression model was 1.213 D [13], which was less accurate than those of the machine learning models (Fig 3).

**Fig 4:**
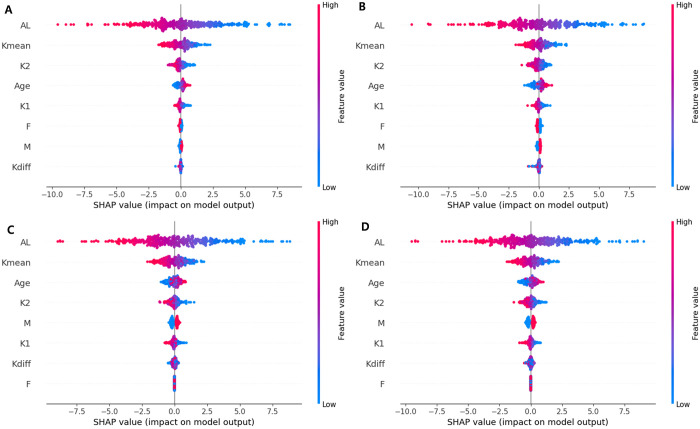
SHAP (SHapley Additive exPlanations) of independent variables (Kdiff, the difference of the keratometry reading; Kmean, mean of keratometry reading; K2, steep K; K1, flat K; AL, axial length; F, female; M, male). Axial length was the most important variable followed by mean keratometry reading to predict spherical equivalents in all models.

**Fig 5:**
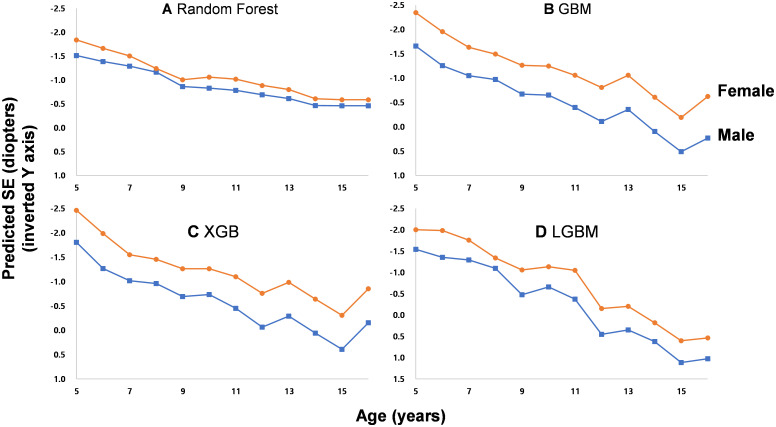
Sex difference of the predicted spherical equivalents (SE) according to age by four different models, with fixed axial length and keratometry readings as the mean of total subjects. Female was predicted to be more myopic than male. As age increased, predicted SE tended to decrease in both groups.

**Fig 6:**
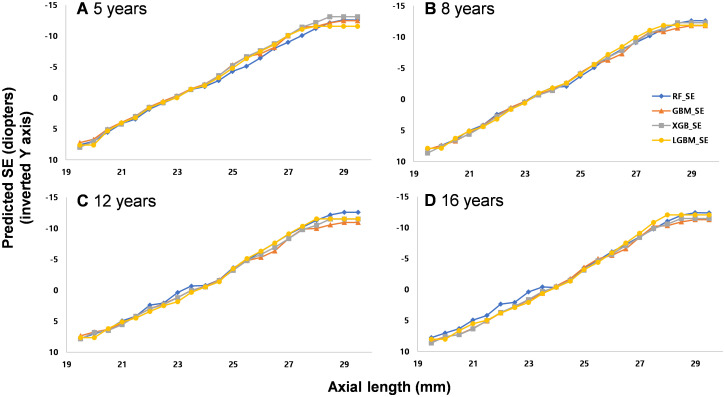
Predicted spherical equivalents (SE) of males according to axial length by four different models in four different age groups (5, 8, 12, and 16 years), with fixed age and keratometry readings as the mean of the total subjects. As axial length increased, the myopic increase was dampened in all age groups.

**Fig 7:**
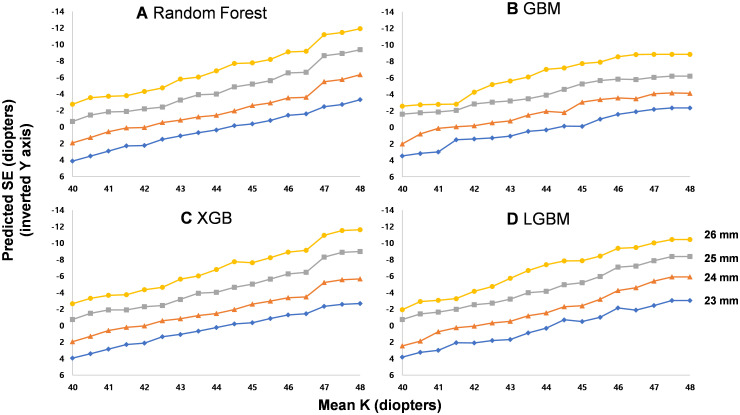
Predicted spherical equivalents (SE) of males according to mean keratometry reading (mean K) in four different models with fixed age and keratometry readings as mean of the total subjects and designated axial lengths (23–26 mm). An almost linear relationship between the myopic SE change and mean K increase was noted.

The SHAP (SHapley Additive exPlanations)of the independent variables is depicted in Fig 4. In the RF, GBM, and XGB models, axial length was one of the most important variables, followed by mean keratometry readings. Using the generated machine learning models, predicted SEs were demonstrated in several presumed clinical circumstances. For this demonstration, the mean value of each variable was used, excluding the variables compared. On comparing the effects of sex differences on SE, female sex was predicted to be more associated with myopic refraction than males with the same axial length and keratometry readings (Fig 5). As axial length increased, the predicted SE was less myopic with other ocular biometric variables fixed (Fig 6). On fixing the difference in keratometry readings at 1.81 (mean of this study population), increasing keratometry readings resulted in a predicted SE that was more myopic (Fig 7).

## Discussion

In this study, we generated an SE prediction model using ocular biometry measurements in childhood, including age and sex. The machine learning models showed more accurate predictions than those of a previous conventional multiple linear regression model. Axial length was the most important variable to predict SE. Even with the same ocular biometry measurements, females were predicted to have more myopia than males. Additionally, older children had a lower myopic refraction than younger children with the same axial length and keratometry readings.

The refractive index of an eye may vary according to several features – such as sex, age, and ocular volume — as the density of the refractive media of an eye may change. To calculate refractive errors using only limited ocular metrics, we should make different relations among ocular metrics considering these features. It is difficult to arbitrarily decide on optimal thresholds for precise modeling. An ensemble regression model created using a machine learning algorithm is a supervised, nonlinear regression model with automated categorization using diverse classifiers that can identify a set of categories an observed variable belongs to, based on diverse loss functions. The ensemble regression model generates different linear regression models for each preclassified group; therefore, an ensemble regression model is more adaptable to real-world multifactorial data, and superior to conventional linear regression for prediction.

In this study, ensemble prediction models predicted more accurately than a conventional linear regression model with an RMSE difference of about 0.4 D. The RMSEs of ensemble models were almost similar, while boosting models (GBM, XGB, LGBM) tended to be slightly more accurate than the bagging model (RF). Boosting models improve their predictive performance by sequentially updating the weights of variables. They have higher predicting accuracy and simultaneously, inevitably higher overfitting problems compared with bagging algorithms. Therefore, the models generated in this study need to be validated by confirming their performance using another external dataset.

Females tended to be more myopic in the simulations, even with the same biometric and clinical variables. Many previous studies reported similar sexual differences. Zadnik et al. [5] reported that females with a shorter axial length were more myopic than males with a longer axial length in a school-based study including school children of multiple ethnicities, aged between 6 and 13 years; however, the females had higher corneal power. Li et al. [14] reported similar results in a Chinese population-based study in children. The study suggested that females had shorter axial lengths, stiffer corneal curvatures, and higher myopic refractions than males in the same age group. Our study demonstrated the same findings using a machine learning model. Additionally, the model demonstrated simulated SEs with designated clinical parameters.

The SE change per unit axial length decreased as axial length increased; a 1 mm increase in axial length induced about −2.2– −2.3 D of SE change. However, the increase in predicted SE was dampened over 27 mm of axial length. A previous study suggested that the refraction to axial length ratio would be dampened as axial length increased based on the calculations of optical theory in Gullstrand’s reduced eye model [15]. By definition, the amount of refractive error of an eye is inversely proportional to the axial length. Therefore, the idea of refraction to axial length dampening is reasonable based on optical principles. This study’s prediction model seemed to reflect such conditions well.

This study should be viewed considering its limitations. Ocular parameters — such as corneal thickness, corneal topography, anterior chamber depth, pupil size, lens curvature/thickness, and refraction test variability (variability caused by examiners and examinees) — were not collected in this study, thus limiting the accuracy of the model, with an RMSE of about 0.8 D. Additional data on ocular parameters can improve the model’s accuracy. Additionally, incorporating various clinical and lifestyle factors beyond biometric features—such as visual acuity, parental history of myopia, prior ocular treatments (e.g., interventions for myopia), and lifestyle information (e.g., time spent outdoors, screen time, and near work duration)—could potentially enhance the model’s predictive accuracy. Although tree-based ensemble models are generally robust in terms of predictive performance, multicollinearity among the variables in this study may still influence both the model’s performance and the interpretation of feature importance. Considering that the machine learning process largely depends on observed data, the model needs external validation despite being confirmed using a split test set independent of the training set.

In conclusion, we generated an ensemble regression model for ocular parameters and refractive errors using the machine learning technique. With this model, refractive errors can be calculated in childhood using only ocular biometric parameters, independent of inevitable compounding factors such as accommodation, examiner’s skill, or examinee’s cooperation. This machine learning model was able to simulate the relationships between ocular parameters and SE, even under hypothetical conditions, to understand the relationships between ocular biometry and refraction.
